# Simple Risk Model Predicts Incidence of Atrial Fibrillation in a Racially and Geographically Diverse Population: the CHARGE‐AF Consortium

**DOI:** 10.1161/JAHA.112.000102

**Published:** 2013-04-24

**Authors:** Alvaro Alonso, Bouwe P. Krijthe, Thor Aspelund, Katherine A. Stepas, Michael J. Pencina, Carlee B. Moser, Moritz F. Sinner, Nona Sotoodehnia, João D. Fontes, A. Cecile J. W. Janssens, Richard A. Kronmal, Jared W. Magnani, Jacqueline C. Witteman, Alanna M. Chamberlain, Steven A. Lubitz, Renate B. Schnabel, Sunil K. Agarwal, David D. McManus, Patrick T. Ellinor, Martin G. Larson, Gregory L. Burke, Lenore J. Launer, Albert Hofman, Daniel Levy, John S. Gottdiener, Stefan Kääb, David Couper, Tamara B. Harris, Elsayed Z. Soliman, Bruno H. C. Stricker, Vilmundur Gudnason, Susan R. Heckbert, Emelia J. Benjamin

**Affiliations:** 1 Division of Epidemiology and Community Health, School of Public Health, University of Minnesota, Minneapolis, MN (A.A.); 2 Department of Epidemiology, Erasmus Medical Center, Rotterdam, The Netherlands (B.P.K., C.W.J., J.C.W., A.H., B.C.S.); 3 Department of Internal Medicine, Erasmus Medical Center, Rotterdam, The Netherlands (B.C.S.); 4 Department of Medical Informatics, Erasmus Medical Center, Rotterdam, The Netherlands (B.C.S.); 5 Icelandic Heart Association, Research Institute, Kopavogur, Iceland (T.A., V.G.); 6 The University of Iceland, Reykjavik, Iceland (T.A., V.G.); 7 Department of Biostatistics, Boston University School of Public Health, Boston, MA (K.A.S., M.J.P., C.B.M.); 8 Department of Epidemiology , Boston University School of Public Health, Boston, MA (E.J.B.); 9 Cardiac Arrhythmia Service, Massachusetts General Hospital, Boston, MA (S.A.L., P.T.E.); 10 Division of Cardiology, Department of Medicine, University of Washington, Seattle (N.S.); 11 Cardiovascular Health Research Unit, Department of Medicine, University of Washington, Seattle (N.S.); 12 Department of Biostatistics, University of Washington, Seattle, WA (R.A.K.); 13 Department of Epidemiology, University of Washington, Seattle, WA (S.R.H.); 14 National Heart Lung and Blood Institute's and Boston University's Framingham Heart Study, Framingham, MA (M.F.S., J.F., J.W.M., D.D.M.M., M.G.L., D.L., E.J.B.); 15 Netherlands Consortium for Healthy Aging (NCHA), The Netherlands (C.W.J., J.C.W., A.H., B.C.S.); 16 Department of Medicine, Boston University School of Medicine, Boston, MA (J.W.M., E.J.B.); 17 Department of Health Sciences Research, Mayo Clinic, Rochester, MN (A.M.C.); 18 Cardiovascular Research Center, Massachusetts General Hospital, Charlestown, MA (M.F.S., S.A.L., P.T.E.); 19 Department of General and Interventional Cardiology, University Heart Center Hamburg‐Eppendorf, Germany (R.B.S.); 20 Department of Epidemiology, Gillings School of Global Public Health, University of North Carolina, Chapel Hill, NC (S.K.A.); 21 Department of Biostatistics, Gillings School of Global Public Health, University of North Carolina, Chapel Hill, NC (D.C.); 22 Division of Public Health Sciences, Wake Forest School of Medicine, Winston‐Salem, NC (G.L.B.); 23 Laboratory of Epidemiology, Demography, and Biometry, National Institute of Aging, National Institutes of Health, Bethesda, MD (L.J.L., T.B.H.); 24 Center for Population Studies, NHLBI, Bethesda, MD (D.L.); 25 Division of Cardiology, University of Maryland Medical Center, Baltimore, MD (J.S.G.); 26 Department of Medicine I, University Hospital Munich, Campus Grosshadern, Ludwig‐Maximilians University, Munich, Germany (M.F.S., S.); 27 Epidemiological Cardiology Research Center (EPICARE), Department of Epidemiology and Prevention, Wake Forest University School of Medicine, Winston‐Salem, NC (E.Z.S.); 28 Inspectorate for Health Care, The Hague, The Netherlands (B.C.S.); 29 Munich Heart Alliance, Munich, Germany (S.); 30 Department of Medicine and Quantitative Health Sciences, University of Massachusetts, Worcester, MA (D.D.M.M.); 31 Department of Biomedical Engineering, Worcester Polytechnic Institute, Worcester, MA (D.D.M.M.)

**Keywords:** atrial fibrillation, epidemiology, risk factors

## Abstract

**Background:**

Tools for the prediction of atrial fibrillation (AF) may identify high‐risk individuals more likely to benefit from preventive interventions and serve as a benchmark to test novel putative risk factors.

**Methods and Results:**

Individual‐level data from 3 large cohorts in the United States (Atherosclerosis Risk in Communities [ARIC] study, the Cardiovascular Health Study [CHS], and the Framingham Heart Study [FHS]), including 18 556 men and women aged 46 to 94 years (19% African Americans, 81% whites) were pooled to derive predictive models for AF using clinical variables. Validation of the derived models was performed in 7672 participants from the Age, Gene and Environment—Reykjavik study (AGES) and the Rotterdam Study (RS). The analysis included 1186 incident AF cases in the derivation cohorts and 585 in the validation cohorts. A simple 5‐year predictive model including the variables age, race, height, weight, systolic and diastolic blood pressure, current smoking, use of antihypertensive medication, diabetes, and history of myocardial infarction and heart failure had good discrimination (C‐statistic, 0.765; 95% CI, 0.748 to 0.781). Addition of variables from the electrocardiogram did not improve the overall model discrimination (C‐statistic, 0.767; 95% CI, 0.750 to 0.783; categorical net reclassification improvement, −0.0032; 95% CI, −0.0178 to 0.0113). In the validation cohorts, discrimination was acceptable (AGES C‐statistic, 0.664; 95% CI, 0.632 to 0.697 and RS C‐statistic, 0.705; 95% CI, 0.664 to 0.747) and calibration was adequate.

**Conclusion:**

A risk model including variables readily available in primary care settings adequately predicted AF in diverse populations from the United States and Europe.

## Introduction

Atrial fibrillation (AF), a common cardiac arrhythmia, has emerged as a major public health problem as a result of wide prevalence,^1^ close relation to stroke and mortality,^2^ and associated costs.^3^ Tools for the prediction of AF could help identify high‐risk individuals and serve as a benchmark to test potential novel risk factors. To this end, the Framingham Heart Study (FHS) developed a risk score for AF, which included a number of variables easily obtained during routine clinical examination.^4^ This risk score was recently validated in 2 additional population‐based cohorts, the Age Gene/Environment Susceptibility‐Reykjavik (AGES) Study and the Cardiovascular Health Study (CHS), where it demonstrated reasonable performance.^5^ An alternative score has been developed in the Atherosclerosis Risk in Communities (ARIC) Study, with similar predictive capability.^6^ These studies included atrial flutter in their definition of AF. This inclusion is reasonable because, even though atrial flutter and AF are electrophysiologically distinct, most patients with atrial flutter have or will develop AF and the risk of stroke associated with atrial flutter is similar to that observed in AF.^7–8^

Previous risk models are limited as a result of being developed in single cohorts. Though the FHS risk score has predicted AF reasonably well in other populations,^5–6^ it is unknown whether a risk model developed in a more geographically or racially diverse population would better predict AF. Previously developed models also require information from a 12‐lead electrocardiogram, which might be unavailable in some primary care settings. Therefore, we developed and validated a new predictive score for AF (including atrial flutter) in 5 US and European cohorts participating in the *C*ohorts for *H*eart and *A*ging *R*esearch in *G*enomic *E*pidemiology (CHARGE) AF consortium.^9^

## Methods

### Study Cohorts

Participant‐specific data from 3 community‐based cohorts in the United States (ARIC, CHS, and FHS) were pooled to develop a risk score for predicting AF, and the validation of this score was performed in 2 additional cohorts in Europe (AGES and the Rotterdam Study [RS]). A brief description of each participating cohort is provided below. For each cohort the determination of which examination to select as baseline was based on the availability of potential predictors and adequate follow‐up for the development of AF. Participants were excluded from this analysis if they had AF at baseline, were younger than 46 or older than 94 years of age, had serum creatinine ≥2.0 mg/dL, identified themselves as other than white or African American (n=30 ARIC, n=32 CHS, and n=62 RS participants), or had missing values for any of the variables of interest. After applying exclusion criteria, the derivation cohort included 18 556 participants and the validation cohorts included a total of 7672 participants. The number of individuals excluded by cohort is provided in the Table S1. Institutional Review Boards at the participating institutions approved the individual studies and study participants provided written informed consent.

#### Atherosclerosis Risk in Communities Study

The ARIC study recruited 15 792 men and women, aged 45 to 64 years, from 4 communities in the United States (Forsyth County, NC; Washington County, MD; Jackson, MS; and suburbs of Minneapolis, MN) in 1987–1989.^10^ Participants were mostly white in the Minnesota and Washington County field centers, white and African American in Forsyth County, and exclusively African American in the Jackson field center. After study inception, participants had 3 follow‐up examinations, each ≈3 years apart. For the present analysis, we included individuals attending the last follow‐up examination (visit 4, conducted in 1996–1998, n=11 656), with this examination used as baseline in all models. Of these, 10 675 met inclusion criteria.

#### Cardiovascular Health Study

In 1989–1990, CHS recruited 5201 men and women 65 years or older from 4 communities (Forsyth County, NC; Washington County, MD; Sacramento County, CA; and Pittsburgh, PA). Because of the different age inclusion criteria there was no overlap in ARIC and CHS participants. In 1992–1993, 687 African Americans were recruited in 3 of the 4 communities to increase minority representation.^11^ CHS participants had annual follow‐up examinations through 1999 with ongoing surveillance for cardiovascular events from baseline through the present. The 1989–1990 examination was considered baseline for 3768 (≈65%) of the eligible CHS participants in this analysis, while 1992–1993 was the baseline examination for the rest (n=1275).

#### Framingham Heart Study

In 1971–1975, the FHS Offspring cohort recruited 5124 predominantly white men and women, offspring (and their spouses) from the Original FHS cohort with follow‐up examinations every 4 to 8 years.^12^ The current analysis included participants of the FHS Offspring cohort free of AF attending the 6th examination cycle (1995–1998, n=3113); 2838 met inclusion criteria and were included in the analysis.

#### Age, Gene/Environment Susceptibility Reykjavik Study

The original Reykjavik Study, conducted between 1967 and 1996, included ≈19 000 men and women living in the greater Reykjavik area, born between 1907 and 1935.^13^ Survivors of this study were invited to be part of AGES, which recruited 5764 men and women in 2002–2006. Of these, 5427 had a complete clinic exam, and 4469 met inclusion criteria and were considered for this analysis.

#### Rotterdam Study

The RS, a prospective population‐based study aimed to assess the determinants of chronic conditions in the elderly, examined 7983 men and women, aged 55 years and older, living in the Rotterdam suburb of Ommoord in 1989–1993.^14^ Since then, participants have been continuously followed and were reexamined in 1993–1994, 1997–1999, 2002–2004 and 2008–2010. The present analysis included 3203 study participants examined in 1997–1999 meeting inclusion criteria.

### Ascertainment of Incident AF

Incident AF cases in all 5 studies were ascertained from study electrocardiograms and hospitalization discharge diagnosis codes (ICD9‐CM 427.3, 427.31 or 427.32, or ICD10 I48 in any position).^15–18^ Individuals with atrial flutter were included as AF cases. AF ascertainment in FHS required additional adjudication of cases by study cardiologists using electrocardiographic and clinical data from the FHS clinic, outside hospital, or general practitioner records.^17^ Cases included in the present analysis occurred between 2002–2011 in AGES, 1996–2005 in ARIC, 1989–2000 in CHS, 1995–2005 in FHS, and 1997–2005 in RS. Further details of AF ascertainment are available in the online supplementary materials.

### Other Measurements

In all 5 study cohorts, examinations included a 12‐lead electrocardiogram, standardized measurements of anthropometry, blood pressures, blood lipids, and fasting glucose, as well as assessment of prior cardiovascular disease and medication use.^10–13,19^ Details on measurement methods are provided in the online supplementary materials. Protocols for variable ascertainment and definitions of cardiovascular risk factors were comparable across cohorts.

### Statistical Analysis

#### Derivation of the predictive model

Means and standard deviation and frequency distribution of relevant covariates were calculated by cohort and race. We initially ran cohort‐ and race‐specific Cox proportional hazard models to assess individual predictors of AF after age‐ and sex‐adjustment in each cohort up to 7 years of follow‐up. Variables considered included age, sex, height, weight, current smoking, systolic and diastolic blood pressure, use of antihypertensive medication, history of diabetes, fasting blood glucose, estimated glomerular filtration rate (eGFR) <60 mL/kg per m^2^,^20^ total blood cholesterol, HDL cholesterol, triglycerides, heart rate, electrocardiographic‐derived left ventricular hypertrophy, PR interval, history of coronary artery bypass graft (CABG), history of heart failure, history of myocardial infarction, and history of stroke. We selected as candidate predictors for our pooled model any variable significantly associated with AF (*P*<0.05) in at least 2 of the 3 cohorts, and ran the final Cox proportional hazards model on our participant‐specific pooled data using backward selection of variables (*P*<0.05 to remain in the model). Age, sex, and race interactions were tested, as was the assumption of proportional hazards. Model‐based individual 5‐year risk of AF was calculated. We evaluated model performance using the C‐statistic,^21^ discrimination slopes,^22^ and Nam and D'Agostino's modified Hosmer‐Lemeshow chi‐square statistic for survival analysis.^23^

To facilitate the use of our score in those clinical settings with limited access to electrocardiograms or blood tests, we first developed a predictive model that did not require information from electrocardiogram and blood tests (which we labeled “simple model”). We then developed a more complex model adding electrocardiographic variables and blood tests (labeled “augmented model”). Variables were retained in the models if they were significantly associated with AF incidence (*P*<0.05). We calculated the added predicted value of the augmented model versus the simple model with the increment in the C‐statistic and the categorical net reclassification improvement (NRI) using the following risk categories: <2.5%, 2.5% to 5%, >5%.^22^

#### Validation analysis

The models developed in the derivation cohorts were applied in AGES and the RS to estimate the 5‐year risk of developing AF. As in the derivation analysis, model performance was assessed using the C statistic, discrimination slopes, and Nam and D'Agostino's chi‐square statistic metrics. To improve adjustment fit in the validation cohorts, we accounted for the baseline survival of the respective cohort and the corresponding risk factor means.^24^

#### Additional analyses

We compared the performance of the newly developed risk score with the previous FHS AF risk score.^4^ To this end, we calculated model quality measures in the pooled data from ARIC, CHS, and FHS, and separately in AGES and RS after applying the AF risk function previously derived from FHS.^4^ Because the presence of cardiac murmur, one of the variables included in the FHS AF risk score, was not available in AGES and RS, and given its low prevalence (<3% in the FHS cohort),^4^ we assumed it to be absent for all participants in whom it was not ascertained. Finally, we compared calibration and discrimination of the derived risk model and the model independently derived including those same variables in each validation cohort. SAS‐Software version 9.1 was used for all analyses.

## Results

Baseline characteristics of eligible individuals by cohort and race (in ARIC and CHS) are presented in Table ****1. The average age in years ranged from 60 in FHS to 76 in AGES, and the proportion of women was between 55% and 66% across cohorts. African Americans comprised 19% of the derivation sample. The prevalence of cardiovascular risk factors was generally higher in African Americans than in whites. The analysis included 1186 incident AF cases among 18 556 participants in the derivation cohorts, and 585 cases among the 7672 participants in the validation cohorts.

**Table 1.** Baseline Characteristics in Derivation Cohorts and Validation Cohorts

	Discovery Cohorts					Validation Cohorts	
	FHS	CHS whites	CHS AA	ARIC whites	ARIC AA	AGES	RS
N	2838	4324	719	8305	2370	4469	3203
AF cases	143	560	64	343	76	408	177
Age, y	60 (8)	73 (5)	73 (5)	63 (6)	62 (6)	76 (6)	72 (7)
Sex, % female	54.5	58.8	65.9	54.5	64.4	60.4	58.9
Height, cm							
Women	161 (6)	159 (6)	160 (6)	161 (6)	163 (6)	161 (6)	161(6)
Men	175 (7)	173 (7)	174 (7)	175 (7)	176 (7)	175 (6)	174(7)
Weight, kg	78.3 (17.1)	71.7 (14.3)	77.5 (15.6)	79.7 (16.9)	85.7 (18.2)	75.3 (14.5)	74.4 (12.3)
Current smoker, %	14.6	9.0	14.5	14.6	17.5	12.2	15.6
Systolic blood pressure, mm Hg	130 (19)	136 (21)	142 (22)	126 (18)	134 (20)	142 (21)	143 (21)
Diastolic blood pressure, mm Hg	76 (9)	70 (11)	76 (11)	70 (10)	76 (11)	74 (10)	75 (11)
Antihypertensive medication use, %	29.6	43.8	61.5	29.6	58.4	61.2	37.1
Diabetes, %	10.2	15.0	24.3	10.2	25.9	11.5	10.2
Fasting blood glucose, mg/dL	104 (28)	109 (33)	119 (55)	107 (31)	123 (56)	104 (21)	107 (27)
eGFR <60 mL/kg per m^2^, %	9.1	21.1	25.2	9.1	14.8	28.8	13.4
Total cholesterol, mg/dL	207 (38)	212 (38)	210 (38)	202 (36)	200 (38)	219 (44)	225 (38)
HDL cholesterol, mg/dL	51 (16)	54 (16)	58 (15)	49 (16)	53 (17)	62 (17)	54 (15)
Triglycerides, mg/dL	140 (95)	146 (82)	116 (65)	151 (90)	115 (63)	106 (57)	135 (66)
Heart rate, bpm	64 (10)	65 (11)	67 (14)	62 (10)	65 (11)	66 (11)	68 (11)
Electrocardiogram‐derived LVH, %	0.7	3.5	9.0	0.7	4.8	—	5.0
PR interval, ms	164 (24)	170 (31)	172 (34)	166 (27)	171 (27)	173 (30)	170 (26)
CABG history, %	1.6	4.4	2.8	1.6	1.7	5.9	4.0
Prevalent heart failure, %	0.6	3.6	4.7	3.9	7.2	1.7	3.5
Prevalent myocardial infarction, %	4.0	9.4	8.2	4.0	4.6	7.0	10.8
Stroke history, %	0.5	3.3	5.8	0.5	3.6	6.0	3.7

Values correspond to percent or mean (standard deviation). FHS indicates Framingham Heart Study; CHS, Cardiovascular Health Study; AA, African Americans; ARIC, Atherosclerosis Risk in Communities; AGES, Age, Gene and Environment Reykjavik Study; RS, Rotterdam Study; AF, atrial fibrillation; eGFR, estimated glomerular filtration rate; HDL, high‐density lipoprotein; LVH, left ventricular hypertrophy; CABG, coronary artery bypass graft surgery

A number of sociodemographic variables and cardiovascular risk factors were consistently associated with age‐ and sex‐adjusted AF incidence across cohorts (Table****2). We observed a higher risk of incident AF in men, older individuals, those with higher height, weight, blood pressure, and blood glucose, individuals with lower total cholesterol, with electrocardiographic left ventricular hypertrophy, who use hypertension medication, with diabetes, who are current smokers, and with a previous history of heart failure or myocardial infarction. Alcohol intake was not significantly associated with AF risk in any of the derivation cohorts (data not shown).

**Table 2.** Hazard Ratios (95% confidence intervals) of Atrial Fibrillation by Selected Variables, by Cohort. Models With Individuals Risk Factors Adjusted for Age and Sex

	FHS	CHS whites	CHS AA	ARIC whites	ARIC AA	AGES	RS
Age, per 5 years	1.64 (1.49 to 1.82)	1.49 (1.39 to 1.60)	1.32 (1.09 to 1.63)	1.62 (1.46 to 1.78)	1.54 (1.27 to 1.88)	1.43 (1.31 to 1.56)	1.43(1.29 to 1.59)
Sex, male vs female	1.95 (1.39 to 2.72)	1.87 (1.46 to 2.04)	1.28 (0.77 to 2.13)	1.38 (1.11 to 1.70)	1.21 (0.76 to 1.92)	1.53 (1.26 to 1.86)	1.68(1.25 to 2.27)
Height, per 10 cm	1.25 (0.96 to 1.62)	1.26 (1.11 to 1.44)	1.11 (0.76 to 1.64)	1.39 (1.17 to 1.65)	1.44 (1.08 to 1.91)	1.17 (0.98 to 1.39)	1.41(1.10 to 1.79)
Weight, per 15 kg	1.22 (1.03 to 1.44)	1.18 (1.07 to 1.31)	1.31 (1.04 to 1.65)	1.39 (1.26 to 1.53)	1.23 (1.03 to 1.46)	1.17 (1.04 to 1.31)	1.55(1.28 to 1.87)
Current smoker vs non smoker	1.57 (0.98 to 2.51)	1.35 (1.02 to 1.81)	0.66 (0.28 to 1.53)	1.33 (0.99 to 1.79)	1.27 (0.71 to 2.29)	1.38 (1.04 to 1.83)	0.96(0.62 to 1.50)
Systolic BP, per 20 mm Hg	1.15 (0.97 to 1.37)	1.19 (1.10 to 1.28)	1.15 (0.92 to 1.42)	1.14 (1.02 to 1.28)	0.98 (0.78 to 1.24)	1.09 (1.00 to 1.19)	1.21(1.05 to 1.38)
Diastolic BP, per 10 mm Hg	0.89 (0.75 to 1.06)	1.00 (0.93 to 1.08)	0.90 (0.72 to 1.13)	0.89 (0.80 to 0.99)	1.04 (0.84 to 1.29)	0.97 (0.88 to 1.08)	1.02(0.89 to 1.17)
Antihypertensive medication use	1.82 (1.31 to 2.55)	1.63 (1.38 to 1.92)	1.31 (0.77 to 2.22)	2.02 (1.63 to 2.51)	2.31 (1.34 to 3.98)	1.59 (1.28 to 1.97)	1.62(1.21 to 2.19)
Diabetes	1.63 (1.08 to 2.51)	1.51 (1.23 to 1.86)	1.17 (0.67 to 2.04)	1.83 (1.42 to 2.36)	1.64 (1.03 to 2.62)	1.21 (0.91 to 1.61)	1.23(0.80 to 1.90)
Blood glucose,* per 10 mg/dL	1.05 (1.00 to 1.10)	1.05 (1.02 to 1.07)	1.02 (0.98 to 1.06)	1.06 (1.03 to 1.08)	1.02 (0.98 to 1.05)	1.01 (0.96 to 1.06)	1.06(1.00 to 1.11)
eGFR <60 mL/min per m^2^, vs ≥60	0.66 (0.39 to 1.12)	1.24 (1.02 to 1.50)	1.54 (0.90 to 2.63)	2.05 (1.52 to 2.76)	1.25 (0.71 to 2.19)	1.07 (0.87 to 1.33)	1.17(0.79 to 1.75)
Total cholesterol,* per 40 mg/dL	0.91 (0.76 to 1.10)	0.86 (0.78 to 0.94)	0.91 (0.70 to 1.18)	0.75 (0.66 to 0.85)	0.81 (0.63 to 1.04)	0.89 (0.81 to 0.98)	0.98(0.83 to 1.16)
HDL cholesterol,* per 15 mg/dL	0.86 (0.71 to 1.04)	0.99 (0.91 to 1.08)	1.10 (0.86 to 1.41)	0.82 (0.73 to 0.93)	0.80 (0.63 to 1.02)	0.98 (0.89 to 1.08)	0.86(0.72 to 1.02)
Triglycerides,* per 40 mg/dL	1.02 (0.96 to 1.08)	1.02 (0.98 to 1.07)	0.90 (0.74 to 1.10)	1.03 (0.98 to 1.07)	1.14 (1.04 to 1.26)	1.02 (0.95 to 1.09)	1.09(1.01 to 1.17)
Heart rate, per 10 bpm	0.99 (0.84 to 1.16)	1.05 (0.97 to 1.13)	0.99 (0.82 to 1.19)	1.12 (1.00 to 1.24)	0.99 (0.80 to 1.23)	0.97 (0.89 to 1.06)	0.92(0.80 to 1.06)
LVH by electrocardiogram	2.96 (1.08 to 8.08)	1.91 (1.36 to 2.67)	1.66 (0.82 to 3.37)	2.19 (1.26 to 3.82)	1.52 (0.61 to 3.76)	—	2.44(1.54 to 3.86)
PR interval, per 30 ms	1.03 (0.85 to 1.26)	1.13 (1.05 to 1.22)	1.22 (1.01 to 1.47)	1.05 (0.94 to 1.18)	1.06 (0.83 to 1.35)	1.18 (1.09 to 1.28)	1.04(0.99 to 1.10)
CABG history	0.95 (0.35 to 2.58)	1.84 (1.34 to 2.51)	0.62 (0.09 to 4.45)	2.35 (1.70 to 3.25)	1.32 (0.32 to 5.44)	1.66 (1.18 to 2.35)	1.40(0.75 to 2.60)
Heart failure history	2.08 (0.66 to 6.56)	3.27 (2.44 to 4.39)	3.20 (1.52 to 6.73)	2.57 (1.80 to 3.67)	3.94 (2.30 to 6.75)	2.52 (1.51 to 4.23)	1.45(0.76 to 2.79)
Myocardial infarction history	1.71 (0.99 to 2.96)	2.04 (1.63 to 2.55)	1.57 (0.71 to 3.45)	2.39 (1.72 to 3.32)	2.13 (0.98 to 4.65)	1.43 (1.03 to 1.98)	1.37(0.91 to 2.06)
Stroke history	0.71 (0.10 to 5.11)	2.38 (1.71 to 3.30)	1.91 (0.86 to 4.21)	1.96 (1.12 to 3.42)	1.64 (0.60 to 4.50)	1.01 (0.71 to 1.44)	2.24(1.31 to 3.83)

FHS indicates Framingham Heart Study; CHS, Cardiovascular Health Study; AA, African Americans; ARIC, Atherosclerosis Risk in Communities; AGES, Age, Gene and Environment Reykjavik Study; RS, Rotterdam Study; BP, blood pressure; eGFR, estimated glomerular filtration rate; HDL, high‐density lipoprotein; LVH, left ventricular hypertrophy; CABG, coronary artery bypass graft surgery.

* Fasting.

### Derivation of the Predictive Model

Using a backward‐selection algorithm in pooled data from ARIC, CHS and FHS, the following variables were included in the simple risk prediction score: age, race, height, weight, systolic blood pressure, diastolic blood pressure, current smoking, use of antihypertensive medication, diabetes, history of myocardial infarction, and history of heart failure. In addition to these variables, the PR interval and electrocardiogram‐derived left ventricular hypertrophy were selected to be included in the augmented prediction score. The augmented score did not select variables requiring measurement of lipid levels, blood glucose, or creatinine. No significant interactions with age, sex, or race were observed. Table****3 includes the beta coefficients, standard errors, and hazard ratios with their 95% CIs corresponding to the final simple and augmented predictive models. The simple predictive model achieved good performance (C‐statistic, 0.765; 95% CI, 0.748 to 0.781).

**Table 3.** Final Multivariable Model for 5‐year Risk of AF Derived in ARIC, CHS, and FHS*

Variable	Simple Model		Augmented Model	
	Estimated β (SE)	HR (95% CI)	Estimated β (SE)	HR (95% CI)
Age (5 years)	0.508 (0.022)	1.66 (1.59, 1.74)	0.501 (0.022)	1.65 (1.58, 1.72)
Race (white)	0.465 (0.093)	1.59 (1.33, 1.91)	0.486 (0.094)	1.63 (1.35, 1.95)
Height (10 cm)	0.248 (0.036)	1.28 (1.19, 1.38)	0.243 (0.037)	1.28 (1.19, 1.37)
Weight (15 kg)	0.115 (0.033)	1.12 (1.05, 1.20)	0.121 (0.033)	1.13 (1.06, 1.20)
Systolic BP (20 mm Hg)	0.197 (0.033)	1.22 (1.14, 1.30)	0.186 (0.033)	1.20 (1.13, 1.29)
Diastolic BP (10 mm Hg)	−0.101 (0.032)	0.90 (0.85, 0.96)	−0.098 (0.032)	0.91 (0.85, 0.97)
Smoking (current)	0.359 (0.091)	1.43 (1.20, 1.71)	0.365 (0.091)	1.44 (1.20, 1.72)
Antihypertensive medication use (Yes)	0.349 (0.063)	1.42 (1.25, 1.60)	0.341 (0.063)	1.41 (1.24, 1.59)
Diabetes (Yes)	0.237 (0.073)	1.27 (1.10, 1.46)	0.242 (0.073)	1.27 (1.10, 1.47)
Heart failure (Yes)	0.701 (0.106)	2.02 (1.64, 2.48)	0.678 (0.107)	1.97 (1.60, 2.43)
Myocardial infarction (Yes)	0.496 (0.089)	1.64 (1.38, 1.96)	0.469 (0.090)	1.60 (1.34, 1.91)
LVH by electrocardiogram (Yes)	—	—	0.401 (0.129)	1.49 (1.16, 1.92)
PR Interval (<120 vs 120 to 199)	—	—	0.645 (0.200)	1.91 (1.29, 2.82)
PR Interval (>199 vs 120 to 199)	—	—	0.118 (0.077)	1.13 (0.97, 1.31)

AF indicates atrial fibrillation; ARIC, Atherosclerosis Risk in Communities; CHS, Cardiovascular Health Study; FHS, Framingham Heart Study; SE, standard error; HR, hazard ratio; CI, confidence interval; BP, Blood pressure; LVH, Left ventricular hypertrophy.

* All risk factors are classified at baseline examination. The 5‐year risk for the simple model can be calculated as 1−0.9718412736^exp(ΣβX −12.5815600)^ where β is the regression coefficient and X is the level for each risk factor; the risk for the augmented model is given as 1−0.9719033184^exp(ΣβX −12.4411305)^. When calculating the 5‐year risk, estimated β for age, height, weight, systolic and diastolic blood pressure must be divided by the number of presented units.

The addition of information from the electrocardiogram provided no gain in predictive ability (C‐statistic, 0.767; 95% CI, 0.750 to 0.783). Inclusion of pulse pressure instead of systolic and diastolic blood pressure, or of body mass index or waist circumference instead of weight provided similar results (data not shown). Similarly, the categorical NRI showed that the addition of electrocardiographic variables did not improve the predictive ability of the model (NRI, −0.0032; 95% CI, −0.0178 to 0.0113; Table S2).

**Figure 1.**
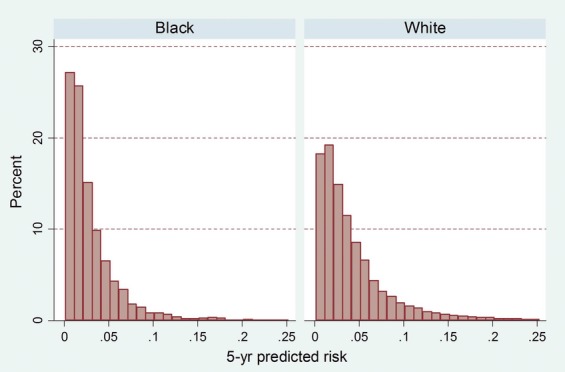
Distribution of predicted 5‐year risk of AF in the derivation cohorts by race using the Cohorts for Heart and Aging Research in Genomic Epidemiology atrial fibrillation (CHARGE‐AF) simple score.

The distribution of predicted 5‐year risk of AF in the derivation cohorts is provided in Figure ****1 and the observed cumulative risk of AF by predicted risk based on the simple model is presented in Figure S1**,** separately for whites and African Americans. An Excel spreadsheet (available as a supplemental file) allows calculation of AF risk using this predictive model.

**Figure 2.**
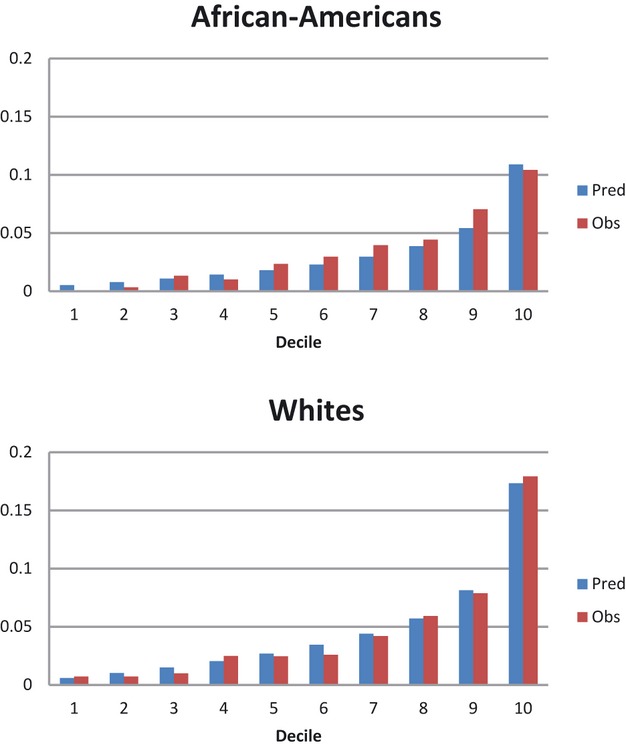
Calibration plots for the Cohorts for Heart and Aging Research in Genomic Epidemiology atrial fibrillation (CHARGE‐AF) simple score model in the combined derivation cohorts, by race. The *x*‐axis refers to deciles of predicted AF risk. Each bar in the graph represents the average observed and predicted AF risk.

Calibration of both models was adequate in the entire derivation sample (Table ****4**,** Figure****2) and individually in each derivation cohort (Table S3). Discrimination using the previously developed FHS AF risk score (C‐statistic, 0.734; 95% CI, 0.717 to 0.750) was lower than with the CHARGE score.

**Table 4.** Model Discrimination and Calibration by Cohort and Risk Score

	Pooled ARIC, CHS and FHS	AGES	RS
N	18 556	4469	3203
CHARGE AF Simple Score			
C‐statistic (95% CI)	0.765 (0.748 to 0.781)	0.664 (0.632 to 0.697)	0.705 (0.663 to 0.747)
Calibration chi‐square (*P*‐value)	9.3 (0.41)	12.6 (0.18)	16.4 (0.06)
Discrimination slope	0.056	0.026	0.022
CHARGE AF Augmented Score			
C‐statistic (95% CI)	0.767 (0.750 to 0.783)	0.665 (0.633 to 0.697)	0.716 (0.680 to 0.761)
Calibration chi‐square (*P*‐value)	5.4 (0.80)	16.7 (0.053)	10.1 (0.34)
Discrimination slope	0.059	0.027	0.023
FHS AF Score*			
C‐statistic (95% CI)	0.734 (0.717 to 0.750)	0.652 (0.621 to 0.684)	0.686 (0.642 to 0.729)
Calibration chi‐square (*P*‐value)	26.5 (0.002)	12.2 (0.20)	8.5 (0.49)
Discrimination slope	0.050	0.025	0.017
Cohort's Own Model			
C‐statistic (95% CI)	—	0.668 (0.637 to 0.700)	0.733 (0.690 to 0.776)
Calibration chi‐square (*P*‐value)	—	11.8 (0.23)	10.9 (0.28)
Discrimination slope	—	0.025	0.026

ARIC indicates Atherosclerosis Risk in Communities; CHS, Cardiovascular Health Study; FHS indicates Framingham Heart Study; AGES, Age, Gene and Environment Reykjavik Study; RS, Rotterdam Study; CHARGE AF, Cohorts for Heart and Aging Research in Genomic Epidemiology atrial fibrillation; CI, confidence interval.

* Discrimination and calibration of FHS AF score were obtained applying the published coefficients and calibrated using overall risk.

### Validation of the Predictive Model

The model developed in ARIC, CHS and FHS, was validated in 2 European cohorts, AGES and RS. Table 4 reports discrimination and calibration of the CHARGE‐AF predictive models in the validation cohorts. C‐statistic values were 0.664 in AGES and 0.705 in RS for the simple model, with similar results for the augmented model. Calibration of the predictive model after recalibration of the model using the average risk in each cohort was adequate in AGES and in RS (Table 4, Figure****2).

In RS, the new CHARGE score performed slightly better than the previous FHS risk score (C‐statistic 0.705 for CHARGE simple score versus 0.686 for FHS score), whereas in AGES the CHARGE and FHS scores had similar discrimination (C‐statistic 0.664 for CHARGE simple score versus 0.653 for FHS score).

Because of the relatively lower discrimination of the predictive model in AGES, we calculated the C‐statistic of a model independently derived in the validation cohorts including the variables selected for the CHARGE risk model. Using this approach, the C‐statistic in AGES was 0.668 (95% CI, 0.637 to 0.700) and in RS was 0.733 (95% CI, 0.690 to 0.776), not very different from values obtained using the CHARGE risk model (Table ****4).

## Discussion

In our individual‐level pooled analysis of 3 large community‐based prospective studies in the United States, we found that a simple risk model including variables routinely collected in a primary care setting are useful to predict the future risk of AF. Discrimination ability of the model was comparable or superior to other risk stratification schemes developed for coronary heart disease or stroke.^24–26^ The predictive model performed reasonably well in 2 additional cohorts in Europe when compared to the cohorts' own models. Including variables obtained from a 12‐lead electrocardiogram provided no significant additional predictive ability.

Previous models for the prediction of AF have been reported already, but these were developed in single cohorts.^4,6^ Although the FHS AF risk score has shown acceptable discrimination in populations other than the cohort in which it was developed,^5–6^ important improvements of the CHARGE‐AF model were the availability of participant‐specific data from several cohorts and the larger sample size included in its development and validation. The CHARGE‐AF model utilized more than 26 000 individuals with over 1750 AF cases. The geographic and racial diversity of the participating cohorts provided increased generalizability over and above the FHS AF risk score alone. A further advantage of the CHARGE‐AF predictive model is that it does not require extra diagnostic tests beyond what is usually available in primary care settings. We also found that the CHARGE‐AF model performed better than the original FHS AF score in the derivation and validation cohorts. However, lack of information on cardiac murmur in ARIC, CHS, RS and AGES limits the value of the FHS AF score in these cohorts. Similarly, we did not study discrimination of the ARIC risk score in the CHARGE cohorts since the ARIC score was derived in a middle‐aged cohort (45 to 64 years old at baseline), whereas most individuals in the present analysis were older.

The CHARGE‐AF predictive model shares some variables with previously developed risk scores for coronary heart disease,^24–25,27^ heart failure,^28–29^ stroke,^26^ or general cardiovascular risk.^30^ However, the weight of individual risk factors in these other models differs from the CHARGE‐AF model and their ability to accurately predict AF has been shown inadequate.^6^

The CHARGE‐AF predictive model could have important research and clinical applications. The most immediate application might be to serve as a standard in evaluating the ability of putative novel clinical factors, biomarkers, subclinical measures, or ‘‐omic’ (eg, genomic, epigenomic, transcriptomic, proteomic, metabolomic) tests to reclassify an individual's risk of developing AF. In addition, the predictive model might be used to select high‐risk individuals for trials of primary prevention of AF or intensive monitoring for AF detection. Our 5‐year predictive model also may be useful once primary prevention strategies are developed, to facilitate identification of individuals more likely to benefit from them. Finally, given the association of some cardiovascular risk factors, such as hypertension, obesity, diabetes, or the metabolic syndrome,^31–35^ with the risk of AF, the CHARGE‐AF predictive model may, in the future, contribute to guidelines for selecting candidates for more aggressive risk factor control. Future randomized trials and observational studies should determine if such approaches are useful and cost‐effective.

In the proposed predictive model we found that higher systolic blood pressure was associated with higher AF risk, whereas diastolic blood pressure was inversely associated with AF incidence. This observation is consistent with a previous report from the FHS in which pulse pressure was a better predictor of AF than systolic or diastolic blood pressure alone.^31^ We chose to include systolic and diastolic blood pressure as separate variables in our model, instead of pulse pressure, because they are more commonly recorded in the clinical setting. Including pulse pressure provided similar results as those presented in the current analyses. Similarly, we included weight in the models even though waist circumference or body mass index, and not weight, may be the pathophysiologically relevant factors. In the derivation cohorts, however, models with waist circumference or body mass index offered similar discrimination ability. Which of these variables is more relevant from an etiopathogenic point of view needs to be addressed in future work.

Several variables included in the CHARGE‐AF predictive model were part of both the published FHS and ARIC AF risk scores, including age, systolic blood pressure, use of antihypertensive medication, and history of heart failure (Table S4). Other variables in the CHARGE‐AF model, however, were part of only one of the risk scores, such as race, smoking, height, diabetes, or myocardial infarction (in ARIC), and body mass index (in FHS). Similar to the ARIC model,^6^ sex was not selected as a predictor in the CHARGE‐AF model. Even though AF incidence is higher in men than women, our model suggests that sex differences in the distribution of AF predictors may account for this disparity. In the initial analysis, we observed an unexpected inverse association between total cholesterol and AF risk. Upon further adjustment, cholesterol levels did not show a significant association with AF. Of note, an inverse association between total and LDL cholesterol was found in an analysis conducted in the ARIC study.^36^

We observed that the model had lower discrimination ability in AGES (C‐statistic, 0.67). Discrimination only minimally improved in a model derived specifically in AGES using the CHARGE‐AF variables (C‐statistic, 0.68). In contrast, discrimination of the CHARGE‐AF model was better in RS (C‐statistic, 0.71). We can only speculate about the reasons to explain these differences. AGES participants were, on average, older than participants from other cohorts. Also, cohort differences in the determination of AF or in the impact of genetic risk factors may partly explain these results.

### Strengths and Limitations

Our work has limitations that must be acknowledged. We restricted the age range of our risk score because very few individuals were younger than 46 or older than 94 years. The applicability of our risk model to individuals <46 or >94 years and to individuals not of African or European ancestry is uncertain. Our risk score will need to be validated outside the United States and Western Europe and in other ethnicities (eg, Asians and Hispanics). Similarly, since participants needed to attend a baseline cohort examination in order to be included,the generalizability of the risk score to hospitalized patients or non‐ambulatory settings is unknown. Most of the cohorts relied on periodic clinic examinations and hospitalization ICD codes leading to the potential for misclassification of AF, though validation studies in the ARIC study, CHS, and other populations have shown adequate validity of this case definition.^15–16,37^ We also have shown previously that age‐ and race‐specific incidence rates of AF in the derivation cohorts were similar in spite of the differences in AF ascertainment.^16^ In addition, we note that AF is not infrequently asymptomatic or paroxysmal, being potentially missed in our cohorts. We included initial, paroxysmal, persistent, and permanent AF for which prediction may be heterogeneous. We acknowledge being unable to accurately comment on risk prediction for AF versus atrial flutter. We combined the 2 for several reasons including that they frequently complicate each other's course,^7^ they are reported to have similar risk factors,^8^ and because ICD codes may not accurately distinguish between them.^38–39^ Furthermore, we did not account for measurement error in determining risk factors. We pooled participant‐level data assuming a priori that the associations of risk factors with AF in the subjects representing 3 large US cohort studies are sufficiently homogeneous. Strengths of our analysis include the large sample size, the number of AF cases included in the analysis, the inclusion of multiple cohort studies—enhancing generalizability, the availability of a large number of possible AF predictors, the racial diversity in the studied samples, and external replication.

In conclusion, we have developed a new risk model for the prediction of AF. The proposed model has the advantage of being simpler, using information readily available in a primary care setting, and having been developed in a larger population. Future research should determine whether biomarkers or genetic factors have value in the prediction of AF beyond that of clinical risk factors.
